# Using a longitudinal multi-method approach to document, assess, and understand adaptations in the Veterans Health Administration Advanced Care Coordination program

**DOI:** 10.3389/frhs.2022.970409

**Published:** 2022-09-09

**Authors:** Marina McCreight, Carly Rohs, Marcie Lee, Heidi Sjoberg, Roman Ayele, Catherine Battaglia, Russell E. Glasgow, Borsika Adrienn Rabin

**Affiliations:** ^1^Denver-Seattle Center of Innovation for Veteran-Centered and Value-Driven Care, Aurora, CO, United States; ^2^VA Eastern Colorado Health Care System, United States Department of Veterans Affairs, Veterans Health Administration, Denver, CO, United States; ^3^Colorado School of Public Health, University of Colorado Anschutz Medical Campus, Aurora, CO, United States; ^4^Adult and Child Center for Outcomes Research and Delivery Science, University of Colorado Anschutz Medical Campus, Aurora, CO, United States; ^5^Department of Family Medicine, University of Colorado Anschutz Medical Campus, Aurora, CO, United States; ^6^Dissemination and Implementation Science Center, UC San Diego Altman Clinical and Translational Research Institute, UC San Diego, La Jolla, CA, United States; ^7^Herbert Wertheim School of Public Health and Human Longevity Science, UC San Diego, La Jolla, CA, United States

**Keywords:** adaptation, RE-AIM framework, FRAME, multi-method approach, longitudinal, implementation

## Abstract

**Background:**

Understanding adaptations supports iterative refinement of the implementation process and informs scale out of programs. Systematic documentation of adaptations across the life course of programs is not routinely done, and efficient capture of adaptations in real world studies is not well understood.

**Methods:**

We used a multi-method longitudinal approach to systematically document adaptations during pre-implementation, implementation, and sustainment for the Veteran Health Administration (VA) Advanced Care Coordination program. This approach included documenting adaptations through a real-time tracking instrument, process maps, Implementation and Evaluation (I&E) team meeting minutes, and adaptation interviews. Data collection was guided by the Reach, Effectiveness, Adoption, Implementation, and Maintenance (RE-AIM) enhanced framework for reporting adaptations and modifications to evidence-based interventions (FRAME) model. Adaptations were evaluated across 9 categories, and analytic team consensus and member-checking were used to validate the results.

**Results:**

A total of 144 individual adaptations were identified across two implementation sites and the four data sources; analytic team consensus and member-checking processes resulted in 50 unique adaptations. Most adaptations took place during the early implementation and mid-implementation phases and were: 1) planned; 2) made to address changes in program delivery; 3) made to extend a component; 4) related to the core component of the intervention concerning notification of the community emergency department visit; 5) initiated by the entire or most of the I&E team; 6) made on the basis of: pragmatic/practical considerations; 7) made with an intent to improve implementation domain (to make the intervention delivered more consistently; to better fit the local practice, patient flow or Electronic Health Record (EHR) and/or for practical reasons); 8) a result of internal influences; 9) perceived to impact the RE-AIM implementation dimension (consistent delivery of quality care or costs). I&E team meeting minutes and process maps captured the highest numbers of unique adaptations (*n* = 19 and *n* = 13, respectively).

**Conclusion:**

Our longitudinal, multi-method approach provided a feasible way to collect adaptations data through engagement of multiple I&E team members, allowing and a broader understanding of adaptations that took place. Recommendations for future research include pragmatic assessment of the impact of adaptations and meaningful data collection without overburdening the implementing teams and front-line staff.

## Introduction

Adaptations, defined as changes to an intervention or implementation strategy to increase fit to the context, are common, expected, and often necessary for the successful uptake and initial, ongoing, and sustained implementation of a program in a real-world setting (1–3). Understanding what adaptations are made at different points in the implementation process can support iterative refinement of the implementation process, enhance interpretation of findings, and inform future scale up of the program in different settings. Systematic documentation of adaptations is not routinely done, and how to capture adaptations in complex studies is still not well understood. While frameworks exist to guide the process of adaptation (4) and to provide a nomenclature of the type of adaptations to interventions and implementation strategies (5, 6), there is less guidance on how to collect data about the adaptations and how to analyze them in terms of frequency, timing, nature, and their potential impact. While there is increasing consensus that more than one method should be used to capture adaptations (2, 7), it is less clear what combination of methods for documenting adaptations yields the most efficient, informative and meaningful information of adaptations. Finally, there is especially little guidance on how to assess the impact of adaptations on diverse implementation outcomes. The Model for Adaptation Design and Impact (MADI) (8) provides a conceptual model to structure adaptations and link them with possible outcomes. However, MADI has not been broadly operationalized and used in studies.

There has been little work done using multiple assessment methods and even fewer comparing more than two methods or presenting data on the types and frequency of adaptations across the life course of an intervention. Our team developed a multi-method approach to documenting adaptations across five research projects, which includes real-time ongoing tracking of adaptations and semi-structured stakeholder interviews to identify changes to an original intervention or implementation strategy (9). We already reported the analysis and findings from one of the five research projects in a separate publication (7). The purpose of the current paper is to expand upon this earlier publication by 1) explicitly focusing on the types, nature, and frequency of adaptations longitudinally; 2) discussing the strengths and limitations of different adaptation assessment methods; 3) presenting specifics about the use of process mapping to assess adaptations; and 4) recommending specific directions for future research and pragmatic use of adaptation methods.

## Methods

We used a longitudinal multi-method approach to systematically collect information about adaptations during the pre-implementation, implementation, and sustainment phases for the Veterans Health Administration (VA) Advanced Care Coordination program and analyzed these data to explore the type of adaptations that were made across time points.

### Intervention

The Advanced Care Coordination (ACC) program was designed to address the care coordination needs of Veterans seeking emergency care in a community emergency department (ED) with a specific focus on social determinants of health (SDOH) (10). The program was led by a VA social worker and included four evidence-based core components: 1) notification from a community ED of a Veteran's visit, 2) comprehensive needs assessment addressing SDOH, 3) individualized clinical interventions and 4) warm handoff to the Veteran's assigned VA primary care team (7, 10). The protocol and initial results have been previously published (11, 12). Intervention implementation period was funded for 3 years at site A and for 1 year at site B.

### Settings

ACC was developed and initially tested at one VA Medical Center (VAMC) in the Rocky Mountain Region with a goal of subsequent expansion (site A). After initial success, the program was expanded to the second site, a VAMC in the Midwest Region (site B). According to the VA system organization of hub and spokes, a VAMC is a large urban medical center, offering primary and multiple specialty care services, both on inpatient and outpatient bases. Both sites created a new role, the community transitions social worker (CTSW), to deliver the program, who were trained in the clinical components and supported by a site champion and central Implementation and Evaluation (I&E) team in the implementation efforts. CTSWs were active participants in implementing the program, providing clinical guidance, and informing decisions about adapting the program to fit the local context and practices. In addition, because of their proximity to the clinical setting, CTSWs were trained and tasked with documenting and tracking adaptations data in real time. Between the two sites, the I&E teams included two CTSW, principal investigator/champion, site champion, and six implementation support members (administrative, analytical, and clinical experts). The I&E team at site B was embedded within the operational partner's office.

### Data collection and sources

We used a pragmatic definition of adaptations to determine which changes should be considered and documented as an adaptation. Adaptations were defined as any changes to the program (intervention or implementation strategy), context that have a potential impact on: 1) implementation, service, and/or clinical outcomes; 2) how the program is being implemented in the current setting (i.e., iterative improvements); 3) the likelihood that the program will: a) continue to be offered at the current setting; b) have sustained impact on outcomes of interest; and/or c) be adopted by other settings. In addition, we also documented changes to the research and evaluation methods. Adaptations data collection and documentation were guided by the Reach, Effectiveness, Adoption, Implementation, and Maintenance (RE-AIM) framework enhanced Expanded Framework for Reporting Adaptations and Modifications developed in our previous work (5, 9, 13).

Guided by this framework, we documented adaptations using the following categories: 1) whether changes were planned or unplanned (we defined planned adaptations those that happened as a result of the discussion with the I&E team); 2) elements of change (e.g., the setting, the format, personnel involved, etc.); 3) type of change (e.g., tailoring to individuals, adding a component, etc.); 4) which core component of the program was affected by the change (e.g., initial notification); 5) roles which initiated the change (e.g., entire or most of team, researcher, etc.); 6) basis for change (e.g., based on our vision or values, based on a framework, etc.); 7) reasons for change (e.g., to increase the number or type of patients contacted, etc.); 8) whether changes were made as a response to external factors or internal issues, 9) the short-term impact of the change as it relates to RE-AIM outcomes; and 10) timing for adaptation (e.g., pre-implementation). The documentation instrument is available in Appendix 1. Adaptations were documented using a multi-method longitudinal approach and included: real time tracking, process maps, I&E team meeting minutes, and adaptations interviews.

#### Real-time tracking

Real-time tracking was accomplished using an Excel-based instrument that was developed based on the RE-AIM expanded FRAME categories and allowed CTSWs to enter adaptations across the life cycle of the ACC program. CTSWs were trained in person on how to operationalize the various fields of the instrument and guided on which adaptations should be documented. Training included education on FRAME categories and definitions and demonstration of the tracking instrument; it was delivered by the implementation specialist (MM) and took approximately 1 h. The implementation specialist assisted in data collection and was available to answer questions and provide feedback on an on-going basis. Real-time tracking process began during the pre-implementation phase and continued through the completion of the program. Program changes for Site A were documented between April 2018 and May 2020, for Site B—between January 2019 and September 2019. Furthermore, real-time adaptations were discussed during the regularly scheduled I&E team meetings, where additional guidance about tracking adaptations was provided to CTSWs as needed.

#### Process maps

Process maps provided a visual depiction of the ACC workflow. We color-coded process maps based on the core elements of the program as: 1) initial notification of Veteran's community ED visit (blue); 2) comprehensive needs assessment (purple); 3) tailored clinical intervention informed by the results of needs assessment (green); and 4) warm hand-off to VA primary care team (orange). CTSWs were trained in person in process mapping methods and skills and were tasked to create the initial process maps of the ACC delivery process in their respective sites. The 1-h training was delivered by a Lean Six Sigma Yellow Belt certified implementation specialist (MM), and included content on: importance of understanding a process of interest; approaching process performers for information on specific tasks in the process; specific steps to design a process map, including a demonstration of Microsoft Visio—a software application to construct process maps; application of process mapping in documenting adaptations. The CTSWs designed the initial process maps, which were reviewed and modified by the implementation specialist to comply with the Lean Six Sigma process mapping guidelines (14). Then the CTSWs made new iterations of process maps as adaptations took place (on average, monthly) to reflect the ACC process at each site. Process maps were created and modified using Microsoft Visio application. Process maps were reviewed by the implementation specialist on as needed basis during the implementation process. Additionally, to confirm the process maps, we were able to observe the CTSW process throughout the implementation phases at site A because of the proximity of the I&E team to the implementation setting. We were able to observe the CTSW daily process once at site B during late implementation site visit. ACC end of project final process maps were constructed by the implementation specialist with input from CTSWs, and an example is provided in Appendix 2.

#### Implementation and evaluation team meeting minutes

Implementation and Evaluation (I&E) team meeting minutes were recorded by designated staff during the regularly scheduled I&E team meeting throughout the duration of ACC at both sites. Process changes were a standing agenda item, and any process changes were discussed and agreed upon by the entire or most of the I&E team, including CTSWs. During early implementation phase, I&E team meetings took place weekly; as implementation progressed, the I&E team meetings moved to a bi-monthly and eventually to a monthly occurrence. I&E team meetings occurred in person at site A and virtually with site B.

#### Adaptation interviews

Upon completion of the program funding period at both sites and toward the end of the implementation phase, we interviewed CTSWs, site champions, and members of the I&E team about most impactful adaptations that took place throughout the ACC implementation process. Interview guides were developed based on RE-AIM expanded FRAME constructs (9). Adaptations interviews were conducted by two trained and experiences qualitative analysts (MM, ML) for both sites between August and October of 2020 over the phone and an audio-conferencing platform. Interviews were recorded and transcribed verbatim. The adaptations interview guide is included in Appendix 3.

### Data management and analysis

**Figure 1:**
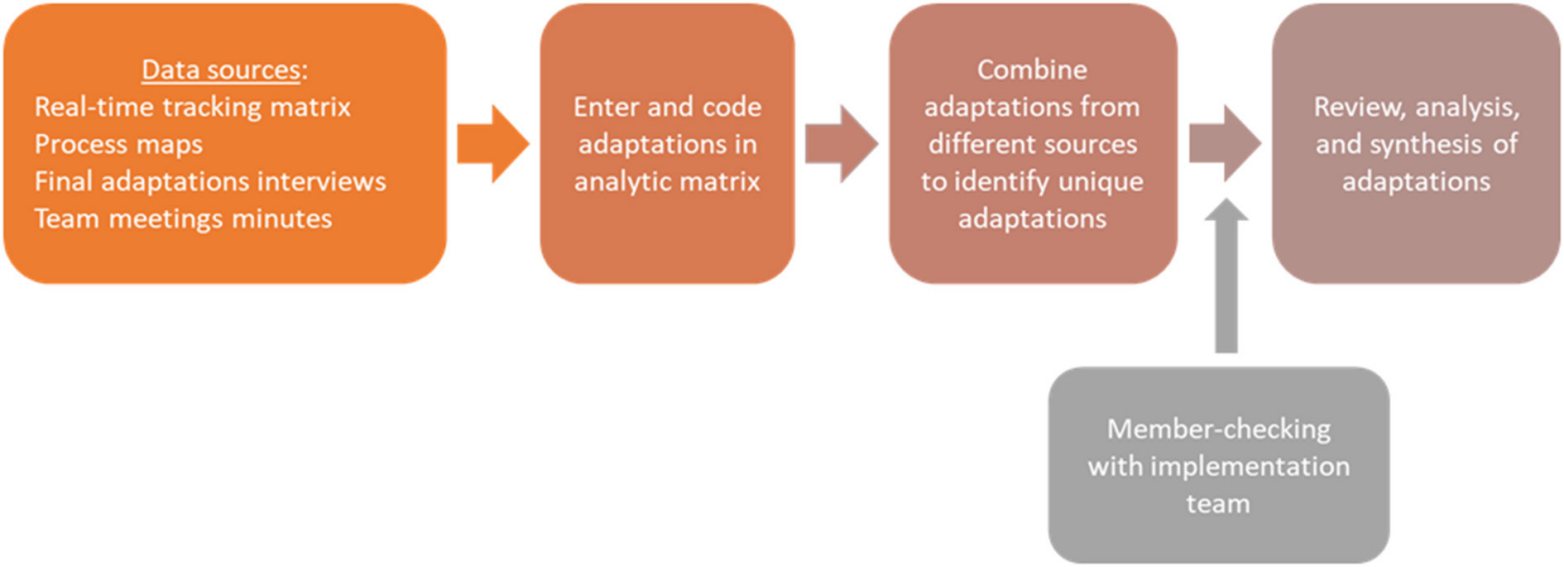
Steps of the analytical process.

The analytical approach for the coding and analysis was adapted from a method developed by one of the sister project team (7) and is outlined in Figure 1. It was based on deductive content analysis with a priori codes. The senior author on this paper (BR) guided the development for these plans and served as a senior implementation scientist on both project analyses. Adaptations data from each source were compiled into a master analytic matrix and then summarized and coded according to the previously described categories; any a posteriori codes for emerging categories were discussed and agreed upon the analytic team. The analyses took place after implementation was competed and was conducted by ACC analytic team members (MM, CR, and BR) and a new team member (ML) who brought unbiased perspectives to the analytical process. The analysts (ML, MM, and CR) cleaned and coded separately the raw data and met to reconcile any coding discrepancies. Specifically, ML and MM coded real-time tracking data. To identify adaptations found within the process maps, analytic team members (MM, ML, CR, and BR) met to compare each iteration of the maps in chronological order. Any change noted from one process map to the next was noted and coded within the FRAME framework. One analyst reviewed all I&E team meetings minutes (MM) and extracted potential adaptations; then the analytic team met to come to a consensus on coding identified adaptations. Adaptations interviews transcripts were reviewed individually by the analysts (MM and ML) who extracted responses into the analytic matrix. The analysts (MM, ML, and CR) came together to discuss similarities and divergences in their coding.

Once all data sets were compiled into the master analytic matrix, each adaptation data entry was assigned a unique identifier number. The next step in the analytical process included identifying unique adaptations across multiple data sources (i.e., multiple entries in the analytic matrix might have described the same adaptation). Thus, the analytic team combined the individual entries to identify unique adaptations to the best of their knowledge of the ACC implementation process and history. To ensure accuracy, member-checking with the CTSWs and other active members of the I&E team was conducted to resolve any questions. The analytic and I&E teams met three times for a total of approximately 3.5 h to discuss questions about adaptations examples, context, and perceived impact, and to validate coding elements, timelines and unique adaptations assignment. Since most members of analytic and I&E team worked closely together (MM and CR were part of the I&E team during implementation), there were no major disagreements. Any uncertainties were related to how we defined categories, and those were flagged and resolved during the member-checking meetings.

Once the analytic matrix entries were confirmed through member-checking, two members of the analytic team developed summary tables by determining the frequency of various types of adaptations and checking for consistency. Individual adaptations from various data sources were combined according to their unique adaptations' assignment. Based on the coded adaptations, data about adaptations were organized across similar themes as described by McCarthy et al. and included in **Table 3** (7). Additionally, we compared the patterns of unique adaptation characteristics across the two sites.

## Results

**Figure 2:**
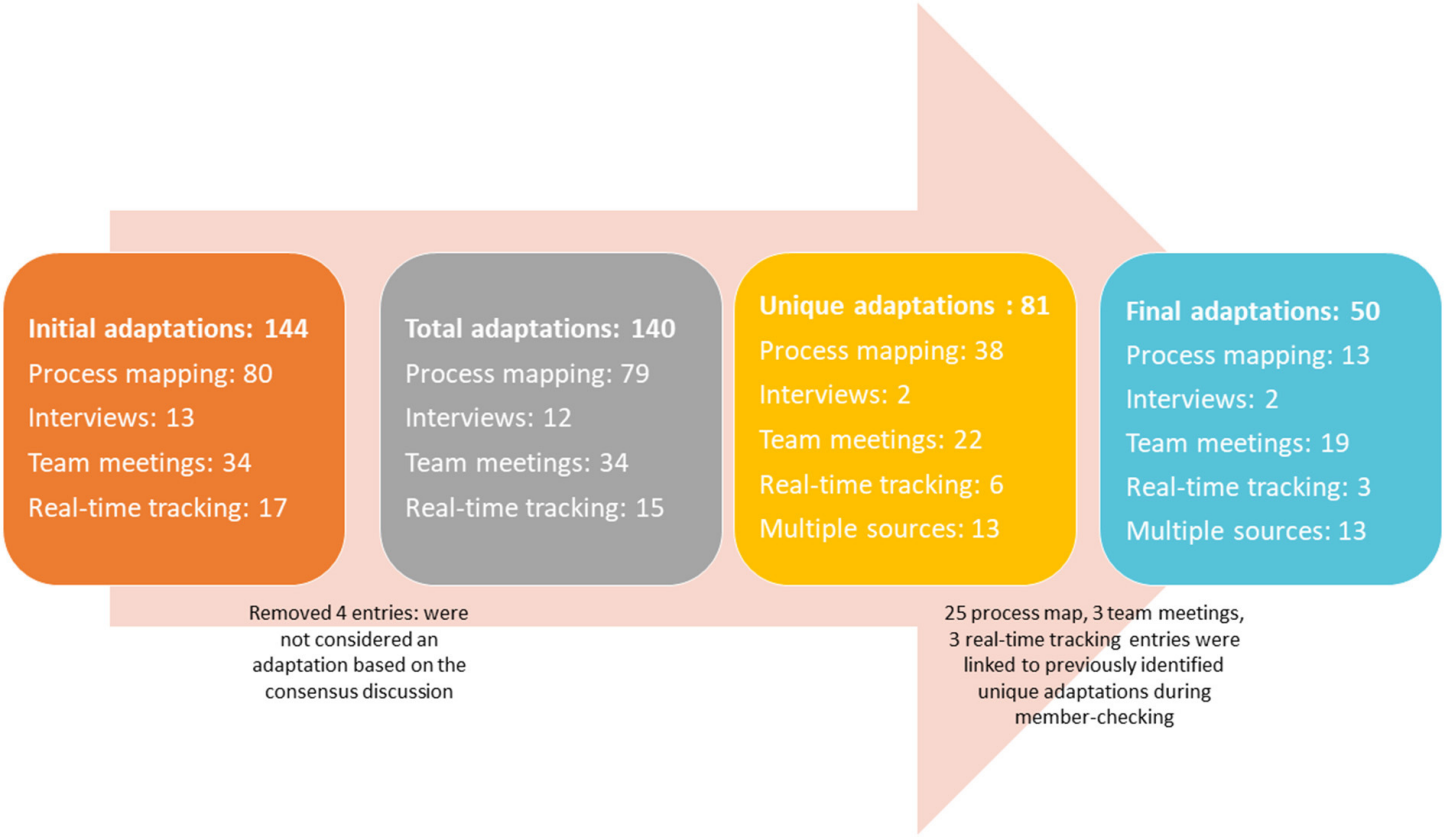
Number of individual adaptation entries and unique adaptations across the two sites and the four data sources.

A total of 144 individual adaptation entries were made across both sites and the four data sources; analytic team consensus and member-checking processes resulted in combining these into 50 unique adaptations. Figure 2 describes how the number of entries and unique adaptations evolved over the course of the data entry, management, and analysis. There were 9 unique adaptations reported by 2 sources, 3 were reported by 3 sources, and 1 was reported by all four sources. Four unique adaptations were reported across multiple time points; for example, on-going changes to the Veteran eligibility criteria was a change that was reported across all implementation phases.

All results are presented from this point on as the number and percent of unique adaptations. Table 1 includes the total number of unique adaptations per site and per implementation phase. There were substantially more unique adaptations in Site A (*n* = 42) than Site B (*n* = 8). At site A, adaptations took place throughout all the implementation phases, with most taking place during early implementation (*n* = 16), and then implementation (*n* = 11), pre-implementation (*n* = 6), late implementation (*n* = 5), and sustainment (*n* = 1); three unique adaptations took place across multiple/all phases. At site B, adaptations took place during early implementation and mid-implementation; one unique adaptation was continuous. Most adaptations took place during implementation (*n* = 4), and then early implementation (*n* = 3); one unique adaptation took place across multiple/all phases. There were no adaptations in the pre-implementation, late- implementation, and sustainment phases in Site B.

**Table 1:** Adaptations identified across time points and sites.

**Site**	**Implementation phase**						
	**Pre-Implementation**	**Implementation**			**Sustainment**	**Across All Phases**	**Total**
		**Early**	**Mid**	**Late**			
Site A	6	16	11	5	1	3	42
Site B	-	3	4	-	-	1	8
Total	6	19	15	5	1	4	50

Table 2 describes the number of unique adaptations captured by each data source. While process mapping captured the most total adaptations (*n* = 80, 55%), the method identified the second largest number of unique adaptations (*n* = 13, 26%). I&E team meetings documented the second largest number of total adaptations (*n* = 34, 24%) and the greatest number of unique adaptations (*n* = 19, 38%). Real-time tracking and interviews captured 17 (12%) and 13(9%) total adaptations, respectively, identifying 3 (6%) and 2 (4%) of unique adaptations, respectively.

**Table 2:** Adaptations from each data source.

**Data source**	**Total adaptations identified**	**Unique adaptations identified**
Process mapping	80	13
Interviews	13	2
I&E Team meetings	34	19
Real-time tracker	17	3

Table 3 describes the types of unique adaptations categorized by the key constructs and response categories of the enhanced FRAME across implementation phases. For each construct we re-coded response categories that were coded initially as “other” to identify emerging subcategories; these were marked as new in the table. Some unique adaptations were categorized using two response categories within a construct (e.g., for the “What elements were changed?” construct an adaptation might have been identified as a change to both the way the program is delivered and how the intervention was presented). As a result, numbers within constructs may not add up to the total number of the unique adaptations (*n* = 50).

**Table 3:** Types of adaptations per enhanced FRAME categories and subcategories.

**Types of adaptations**	**Pre-implementation**	**Implementation**			**Sustainment**	**Across all phases**	**Total**
		**Early**	**Mid**	**Late**			
**Was adaptation planned or unplanned?**							
Planned	6	18	12	3	1	4	44
Unplanned	-	1	3	2	-	-	6
**What elements were changed?**							
The setting	1	-	-	-	-	-	1
The format	-	-	-	-	-	-	-
Personnel involved	-	5	1	4	-	-	10
Target population	-	-	3	-	-	1	4
How intervention is presented	1	-	-	-	-	-	1
Program delivery	4	15	13	2	1	2	37
Refining process map based on input from I&E team^*^	-	-	-	-	-	2	2
Change in implementation strategy^*^	-	1	1	-	-	-	2
**What type of change?**							
Tailoring to individuals	-	-	-	1	-	-	**1**
Adding a component	1	2	2	-	-	-	5
Removing a component	-	-	2	-	-	-	2
Condensing a component	1	1	-	-	-	-	2
Extending a component	1	7	7	1	-	1	17
Substituting for a component	-	-	1	1	-	-	2
Changing the order of components	-	1	-	-	-	-	1
Integrating with other programs we are doing	-	1	2	2	-	-	5
Repeating a component	1	1	-	-	-	-	2
Loosening the structure or protocol	-	-	-	-	-	-	-
Otherwise changing the intervention	-	-	-	-	1	-	1
Changes to recruitment/eligibility criteria^*^	1	3	1	-	-	1	6
Specifying/refining a component^*^	1	3	-	-	-	2	6
Other^*^	1	1	-	1	-	-	3
**To which core component is this change related?** *							
Initial Notification	3	12	10	2	1	4	32
Needs Assessment	3	7	5	1	1	4	21
Clinical Intervention	4	9	4	1	1	4	23
Warm Hand-off to PCP	2	6	1	-	1	4	14
Other	1	2	1	3	-	-	7
**Who initiated this change?**							
Entire or most of the team	5	13	7	3	1	3	32
Practitioner (CTSW)	2	4	7	2	-	1	16
Administrator	-	1	-	-	-	-	1
Researcher	1	4	1	2	-	-	8
Developer	-	-	-	-	-	-	-
Stakeholder	-	1	-	-	-	-	1
Coalition	-	-	-	-	-	-	-
Site Champion^*^	-	1	-	-	-	-	1
Clinical Consultant^*^	-	-	-	1	-	-	1
Implementation Coordinator^*^	-	-	1	-	-	1	2
**On what basis was this change made?**							
Based on our vision or values	-	-	-	-	-	-	-
Based on a framework	-	-	1	1	-	-	2
Based on our knowledge or experience of working with patients	2	8	3	-	-	2	15
Based on QI data, summary information or results	-	-	1	-	-	1	2
Based on pragmatic/practical considerations	3	8	7	3	1	2	24
Based on financial incentives/payment	-	-	1	-	-	-	1
Based on feedback or suggestions	1	5	3	-	-	1	10
Based on our understanding of clinic regulations, procedures and workflow^*^	1	-	1	1	-	-	3
To test a new tool/strategy to inform adaptations^*^	-	1	-	-	-	-	1
**Why was this change made?**							
To increase the number or type of patients contacted (reach)	1	2	3	-	-	1	7
To enhance the impact or success of the intervention for all or important subgroups (effectiveness)	2	9	7	1	-	2	21
To make it possible to involve more teams, team members or staff (adoption)	1	1	-	-	-	-	2
To make the intervention delivered more consistently; to better fit our practice, patient flow or EHR; for practical reasons (implementation)	4	8	8	1	-	3	24
To institutionalize or sustain the intervention (maintenance)	-	-	-	-	1	-	1
To respond to external pressures or policy	-	-	-	2	-	-	2
To save money or other resources (implementation)	-	2	-	1	-	-	3
**Was this adaptation a result of external factors or internal issues?**							
External factors	1	2	3	2	-	-	8
Internal issues	5	17	11	3	1	2	39
Both	-	-	1	-	-	2	3
**What was the short-term impact of this adaptation?**							
No major changes	-	-	2	-	-	-	2
Number or type of patients engaged (Reach)	-	-	4	-	-	-	4
Quality of care or other outcomes (Effectiveness)	-	-	1	-	-	-	1
Participation by teams or staff (Adoption)	1	-	1	-	-	-	2
Consistent delivery of quality care or costs (Implementation)	4	11	7	2	-	4	28
Maintenance or sustainability of the intervention in the practice (Maintenance)	-	2	-	-	-	-	2
Maintenance or sustainability of the patient within the intervention (Maintenance)	-	-	-	-	-	-	-
Reimbursement or financial implications for the practice	-	-	-	-	-	-	-
Efficiency (getting more done faster or with less resources)	-	-	-	-	-	-	-
Unknown	1	6	-	3	1	-	11

* Indicates new category within a construct.

### Was adaptation planned or unplanned?

Most unique adaptations were planned (*n* = 44, 88%), with most planned unique adaptations made during early implementation phase (*n* = 18, 36%) and mid-implementation phase (*n* = 12, 24%). Six unique adaptations (12%) were unplanned, with most taking place in the mid-implementation phase (*n* = 3, 6%). Examples of unplanned unique adaptations in the mid-implementation phase included adding a new referral source for the program: community hospitals needing assistance with enrolling Veterans in the VA's contract nursing home program and coordinating care for Veterans discharging to VA contracted nursing homes. Another example of unplanned unique adaptation was expansion of CTSW role at site B to facilitate care coordination for inpatient referrals due to staffing changes.

### What elements were changed?

Most unique adaptations were involved with the elements of program delivery (*n* = 37, 74%) and personnel involved (*n* = 10, 20%); and most of these took place in the early implementation phase (*n* = 15, 30% and *n* = 5, 10%, respectively). Program delivery adaptations examples included modifications to the eligibility criteria and clarifying the CTSW role to avoid duplication of services provided by existing clinical teams. Example of personnel involved included collaborating with other clinical team members (e.g., specialty clinic social workers) as the implementation progressed. Two additional subcategories emerged after recoding the initial “other” responses: refining process map based on the input from the I&E team and change in implementation strategy.

### What type of change?

Most unique adaptations were made to extend a component (*n* = 17, 34%) in the early implementation and mid-implementation phases (*n* = 7, 14% each). An example of this type of change in the early implementation was extending the CTSW role to notify the Network Authorization Office (NAO) about Veterans' community ED visits. Another example of extending a component occurred during the implementation phase when the role of the CTSW was expanded to include working with inpatient Veterans at the community hospitals to coordinate SDOH-related needs. We created three new subcategories within this construct: 1) Changes to recruitment/eligibility criteria; 2) Specifying/refining a component; and 3) Other.

### To which core component is this change related?

To be able to document which core component was impacted by adaptations, we added a new category to our data collection: the core component of the program to which the change was related. Most unique adaptations made were related to the ACC program Initial Notification (i.e., ways CTSW was notified about a Veterans visit to a community ED) (*n* = 32, 64%), and most of them were made in the early implementation phase (*n* = 12, 24%).

### Who initiated this change?

More than half of all unique adaptations were initiated by the entire or most of the I&E team (*n* = 32, 64%), and most of those took place during the early implementation phase (*n* = 13, 26%). Unique adaptations initiated by the CTSW were the second most frequent (*n* = 16, 32%). Three additional categories were added to clarify the roles that initiated adaptations: Site Champion (*n* = 1), Clinical Consultant (*n* = 1), Implementation Coordinator (*n* = 1).

### On what basis was this change made?

Most changes were made based on pragmatic/practical considerations (*n* = 24, 48%), with most taking place in the early implementation phase (*n* = 8, 16%). An example of adaptation for this category included timing of uploading community ED documentation into the VA electronic medical record. Initially we planned to upload it within a certain time period. However, we learned that community EDs did not always send medical information in timely manner. Therefore, we modified the process to upload the documentation when it was received by the CTSW because of pragmatic/practical considerations.

### Why was this change made?

The reasons for making the adaptation were organized by dimensions that aligned with the various RE-AIM dimensions of reach, effectiveness, adoption, implementation, and maintenance. The intent of most unique adaptations was to improve the Implementation domain of RE-AIM and make the intervention delivered more consistently, improve the fit with practice, enhance patient flow or for practical reasons (*n* = 24, 48%). Most unique adaptations that intended to improve Implementation were made in the early implementation and mid-implementation phases (*n* = 8, 16% each). One example of an adaptation in the early implementation was mailing out the initial Veteran letter with care card immediately after the comprehensive needs assessment instead of mailing it later to provide ACC contact information for Veterans earlier, in case of any repeat ED visits. An example from the mid-implementation phase included adding or removing notification of various clinical team members about Veteran community ED visits. The second largest number of unique adaptations involved an intent to improve the Effectiveness domain of RE-AIM: to enhance the impact or success of the intervention for all or important subgroups (*n* = 21, 42%); most of these unique adaptations with the intent to improve Effectiveness took place during early implementation (*n* = 9, 18%). One example of such an adaptation was the CTSW to follow up with Veterans if they needed additional help from the VA assistance programs in which they were enrolled.

### Was this adaptation a result of external factors or internal issues?

Most unique adaptations were made because of internal issues (*n* = 39, 78%) during early implementation phase (*n* = 17, 34%). Examples of these included changes in the I&E team, collaborating with other VA team members, redefining ACC tasks and specific steps.

### What was the short-term impact of this adaptation?

While we were not able to systematically document the impact of adaptations quantitatively in real-time, we used analytic team consensus and member-checking to retrospectively categorize the adaptations for their short-term impact as perceived by the ACC I&E team members. Of the 50 unique adaptations, 37 adaptations were categorized to impact categories defined by the RE-AIM dimensions. Of the remaining 13 unique adaptations, 2 (4%) were deemed to not have any impact on ACC, and we were unable to determine the impact of the rest 11 (22%) unique adaptations due to the limited recall of the I&E team regarding immediate impacts. Among the unique adaptations that were coded for short-term impact, 28 unique adaptations were indicated to result in improvement in implementation [consistent delivery of quality care or costs (56%)]; 4 unique adaptations impacted reach [number or type patients engaged (8%); 2 impacted adoption (participation by teams or staff (4%)], 2 impacted maintenance [maintenance or sustainability of the intervention in the practice (4%)], and 1 impacted effectiveness [quality of care or other outcomes (2%)].

There were several constructs where FRAME categories were not assigned to the unique adaptations, and these are evident in Table 3. For example, under the “What elements were changed” the format category was not used. Additionally, under the “What type of change” construct the “Loosening the structure or protocol” category was not used.

We compared the patterns of unique adaptation characteristics across the two sites and concluded that generally the patterns between the two sites in terms of the characteristics and types of the adaptations were similar, except for the “Who initiated the change” constructs where the majority of unique adaptations were categorized as “the entire team” for site A (*n* = 32, 76%) and CTSW for Site B (*n* = 8, 100%).

## Discussion

We used a longitudinal multi-method approach to document ACC adaptations in two VA sites across all phases of implementation. A total of 144 individual entries were made concerning adaptations across the two sites and four data sources leading to 50 unique adaptations.

Most unique adaptions to ACC were made during the early implementation phase; we were surprised that no modifications were made during the (early) sustainment phase. We also noted a large difference in terms of both the number and the timing of unique adaptations across the two sites. There were no adaptations documented in the pre-implementation, late- implementation, and sustainment phases and fewer adaptations overall at site B. We suspect several factors that could have attributed to this. First, site A implemented ACC much earlier (almost a year prior), and lessons learned were incorporated when site B began ACC implementation. It also became evident in the late implementation phase that site A would not be sustaining ACC due to divergent leadership priorities. The situation was different at site B—the I&E team was embedded within the operational partner's office and was in proximity to the front-line staff and providers. In addition, it was championed by an operational leader. These factors contributed to the long-term sustainment of ACC at site B—once site B I&E team had an established ACC process, very few modifications were needed to sustain it long-term due to its alignment with leadership priorities and site needs.

Most unique adaptations made were related to the ACC core component I: Initial Notification (how CTSW was notified about a Veteran's community ED visit). This is in line with our experience with implementing ACC: as the program implementation progressed, we were looking to expand ways to receive the notifications. Getting the community EDs staff to notify us of the Veteran ED visit was challenging despite the CTSW contacting them on average twice a week. We were expanding the referral sources and looking for new clinical team members to collaborate with, including various VA clinical care and program office teams. This corresponds with an on-going care coordination issue—how to know that Veterans are receiving care in the community and notify the VA care teams. Currently, there are initiatives and process improvement efforts are being implemented on the system level to address this issue. Another challenge in the care coordination is timing of uploading community ED documentation into the VA electronic medical record. Initially, we planned to upload the received clinical documentation within a certain time period, but we learned that community EDs did not consistently send medical information in timely manner. Therefore, we modified the process to upload the documentation when it was received by the CTSW because of the pragmatic/practical considerations: this was consistent with a finding that transfer of information between VA and community is not consistent, reliable, and does not always take place (10, 15).

Implementation for the ACC was a collaborative approach, and most decisions to make adaptations were made by the entire I&E team as illustrated by the fact that more than half of all unique adaptations were initiated by the entire or most of the I&E team (*n* = 32). Majority of the unique adaptations were made because of internal issues (*n* = 39); examples of these included changes in the I&E team, collaborating with other VA team members, redefining ACC tasks and specific steps. We also noted that most unique adaptations were planned (*n* = 44). As the implementation progressed, the I&E team proactively sought out to make changes to meet the priorities of clinical team members and participating Veterans.

Our findings are consistent and comparable with some of the previously reported work on adaptations to evidence-based health care interventions. Similar to what was reported by McCarthy et al. (7) most of the adaptations were planned—as well as intentional and proactive. At the same time, our results are contrary to some of the previously reported results that describe that most adaptations are not planned and due to external factors and influences. Aschbrenner, for example, describes that most adaptations are not fidelity-consistent, meaning that adaptations take place to modify the original design of an intervention to improve its fit in the real-world context (3). In our experience, most unique adaptations were fidelity-consistent, focused on tailoring to the site context while preserving the core components of ACC. To accomplish that, we trained the site I&E teams on the ACC components and processes and were monitoring the delivery of core components closely at both sites. In addition, we encouraged the site I&E teams to adhere to the original ACC core components described above while adapting their delivery to ensure the fit with the local processes and contexts. External factors (pressures or policies) did not seem to impact the core components in a substantial way, which could explain the fact that most adaptations were planned (3).

Our documentation of the number and type of adaptions across phases of intervention delivery advances the literature. Many studies report on adaptions during the planning or initial stages of a program (e.g., adaptations of a program eligibility criteria), a moderate number during the middle phases of adaption few during the sustainment phase, and to our knowledge almost none across all these phases.

Another contribution of this study was the use of multiple approaches to capture adaptations. Process maps were the largest (*n* = 80) and I&E team meeting minutes were the second largest source of total adaptations (*n* = 34). When identifying unique adaptations, I&E team meetings minutes became the largest source (*n* = 19), and process maps the second largest (*n* = 13) source of data. Real-time tracker and Interviews captured considerably fewer−17 and 13 total adaptations, respectively, identifying 3 and 2 of unique adaptations, respectively. Since interviews took place at the end of the project, we suspect the recall might have been impacted. It was surprising to learn that less than one-third of unique adaptations (*n* = 13) was captured by multiple methods. Moreover, it is curious that few adaptations were identified by more than one method—only 4 of the 50 adaptations were identified by three or more sources, while more were captured by at least two sources (*n* = 9). This potentially speaks to the fact that these methods were focused on capturing changes from different perspectives: i.e., operational perspective (intervention delivery) vs. theoretical (framework-based) approach. We also applied a novel method to document adaptations—the use of process maps, which proved a useful addition to the more typical I&E team meetings adaptation updates. Used alongside other sources of the adaptations data, process maps helped visualize changes taking place across implementation phases. Additional research is needed to understand how it impacts our understanding of adaptations and their effectiveness (16). Although we are proponents of multiple assessments methods, in this study it is questionable whether the interview and real time tracker methods were worth the incremental costs. The interview method may have been more informative and identified more adaptations if it has been conducted at early, mid and late implementation time points rather than only toward the end of the study. We recommend further investigation of the process mapping assessment method to better understand its strengths and limitations. We note parenthetically that use of such maps also lends itself well to assessment of implementation costs.

Determining impact of adaptations was challenging in this project. The questions regarding the impact of the adaptation were the least complete data point and as a result, we were not able to assign short-term impact to 11 unique adaptations. The impact of the 37 unique adaptations was assigned retrospectively during member-checking and could potentially be limited by the recall bias. It is also likely that some period of time is needed for the impact of adaptations to be detected. Additionally, there are few data systems capable of identifying relatively short-term impact of adaptions or to attribute impact to. In the future work, we plan to put processes in place to document short-term impact of adaptations, including examining available data on outcomes and collecting reflections about the impact of the adaptations at 3- and 6-months intervals during the implementation. We also did not assess which combinations of the adaptations that were most likely to lead to sustainment (17).

While the assessment methods used were feasible, relatively comprehensive, and informative, there were limitations in application and interpretation of our approach. We offer the following observations based on our experience assessing and analyzing adaptations using the RE-AIM-expanded FRAME:

1. Adaptations often occurred as a cascade of connected changes in which one change flows into or overlaps with another. As such, adaptations are sometimes not easy to separate into distinct changes and it is important to acknowledge their connections and potential interdependencies when systematically documenting and interpreting them.
2. The sub-categories of adaptations for some of the domains were not well-defined and often did not work well for our documentation purposes, leading to many cases selecting “other” or otherwise changing intervention categories and leading to a further re-coding of these into existing or emerging categories. More specificity for the sub-categories (i.e., definitions) and/or the development of study specific sub-categories could make documentation of adaptations more straightforward.
3. It was challenging to identify roles to fit in the framework categories as people had multiple roles during the implementation—we found ourselves needing to add new roles to capture roles on the I&E team initiating adaptations.
4. Adaptations happened at individual site level or the full research program level. When adaptations were made at one site and then implemented with those changes at the other site, it was challenging to capture these connections.
5. Having a research analyst support the adaptation analysis who was not part of the I&E team provided a helpful and unbiased perspective during the coding process. As someone with an objective perspective, the analyst helped ensure that the categories reflected the data that was presented when there were nuanced iterations of the data. Nevertheless, it was critical to continue checking in with the I&E team for further context for adaptations and validation of coding decisions.

We identified several lessons learned and recommendations for future work documenting adaptations. These include:

1. Establishment of a very early documentation system for adaptations so pre-implementation adaptations are more accurately and comprehensively captured.
2. More streamlined use of adaptation documentation methods that do not place additional burden on the I&E team and frontline providers.
3. Providing standardized, thorough, efficient training to those documenting adaptations to ensure consistent use of adaptation categories.
4. More intentional, pro-active evaluation of the impact of adaptations on both implementation and short- and long-term effectiveness outcomes, while implementation is still taking place.
5. Establishing a process to capture adaptations during the sustainment phase with focus on maintenance. Specifically, identifying changes that inform the long-term sustainment of the interventions and following up with the site I&E teams sometime after implementation is completed.

## Conclusion

The multi-method approach used across multiple time points of the research project proved a feasible way to document adaptations. Triangulation of data from multiple sources increased understanding of adaptations. The approach allowed engagement of multiple I&E team members, which resulted in richer consensus discussions and increased our objectivity. Future work is needed to evaluate the strengths and limitations of various adaptation assessment methods, including pragmatic assessment of the impact of adaptations and meaningful data collection without overburdening the implementing teams and front-line staff and providers.

## Data availability statement

The raw data supporting the conclusions of this article will be made available by the authors, without undue reservation.

## Ethics statement

Ethical review and approval was not required for the study on human participants in accordance with the local legislation and institutional requirements. Written informed consent for participation was not required for this study in accordance with the national legislation and the institutional requirements.

## Author contributions

BR conceptualized tracking instrument and adaptations interview guide. MM, CR, ML, RA, and HS contributed to the data collection and analysis. All authors were involved in project implementation accept ML and contributed to the interpretation of results. MM drafted and compiled the manuscript. All authors provided revisions and feedback on manuscript content.

## Funding

This work was funded by the Veterans Affairs Health Services Research and Development (Grant No. QUE 15-268).

## Conflict of interest

The authors declare that the research was conducted in the absence of any commercial or financial relationships that could be construed as a potential conflict of interest.

## Publisher's note

All claims expressed in this article are solely those of the authors and do not necessarily represent those of their affiliated organizations, or those of the publisher, the editors and the reviewers. Any product that may be evaluated in this article, or claim that may be made by its manufacturer, is not guaranteed or endorsed by the publisher.

## Author disclaimer

The contents of this work are the author's sole responsibility and do not necessarily represent the official views of the VA.

## Supplementary material

Appendix 1 Documentation Instrument: real time tracker (Excel spreadsheet).

Appendix 2 Example of a process map.

Appendix 3 Adaptations interview guide.
