# Supplementary figures and images for: Using a longitudinal multi-method approach to document, assess, and understand adaptations in the Veterans Health Administration Advanced Care Coordination program
Source: Front Health Serv. 2022 Sep 9;2:970409. doi: 10.3389/frhs.2022.970409 (PMC10012685; doi:10.3389/frhs.2022.970409)

## Example of a Process Map

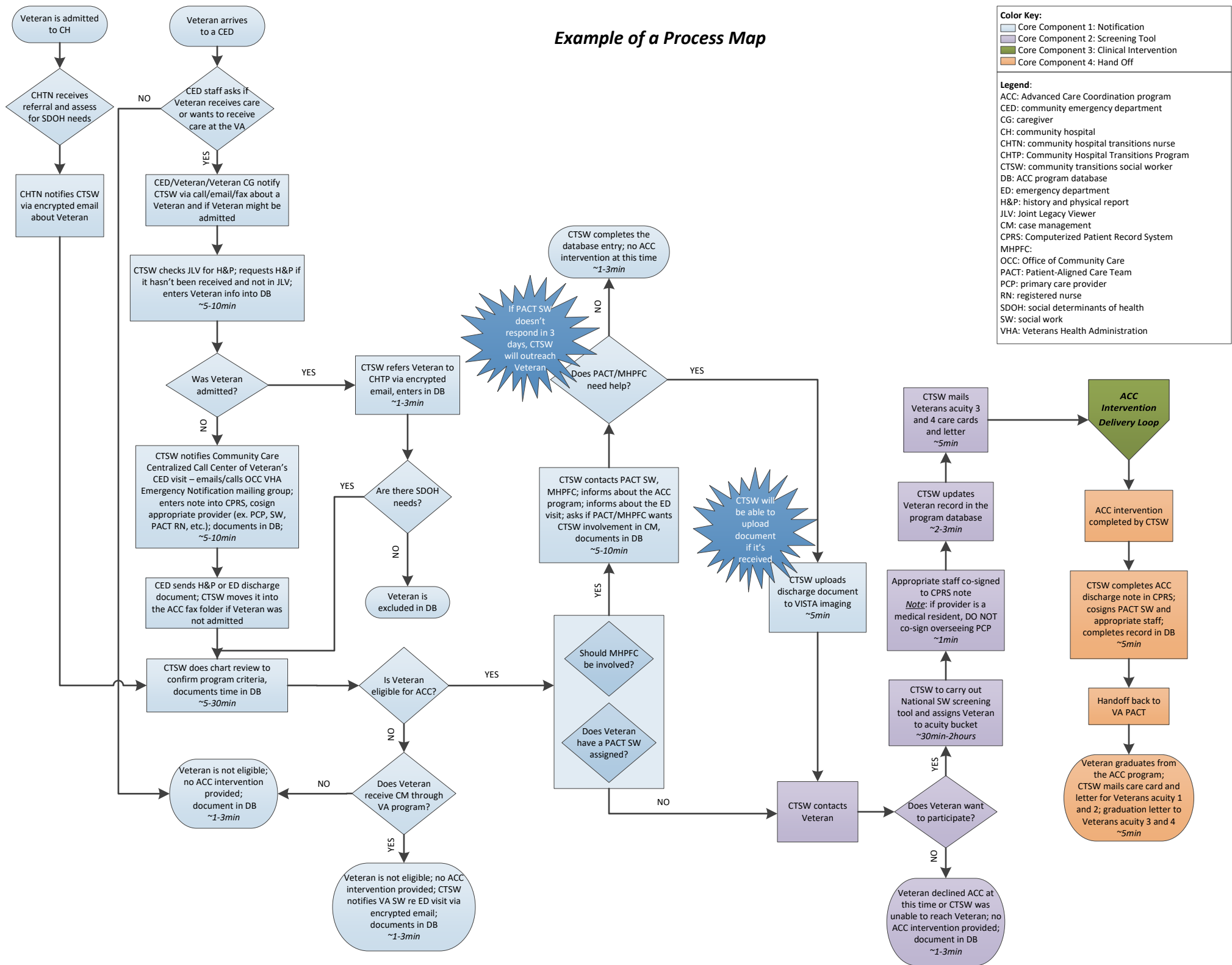

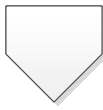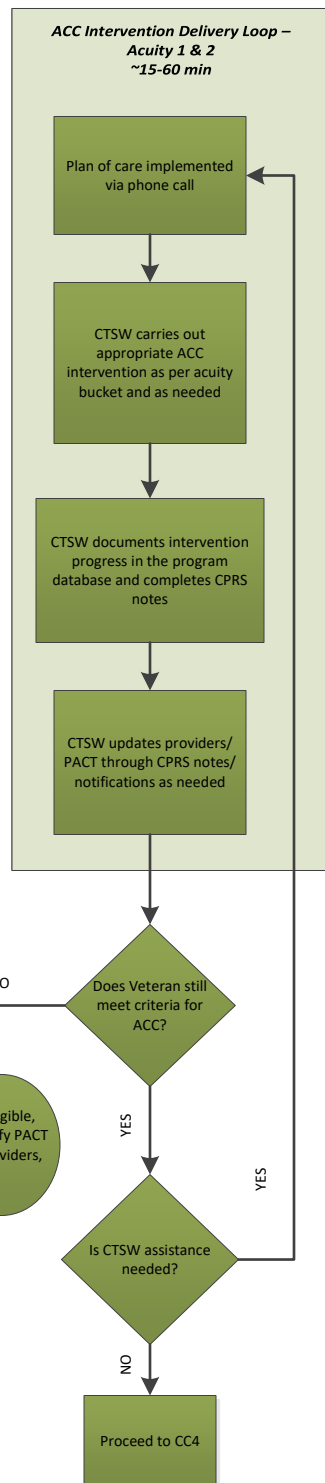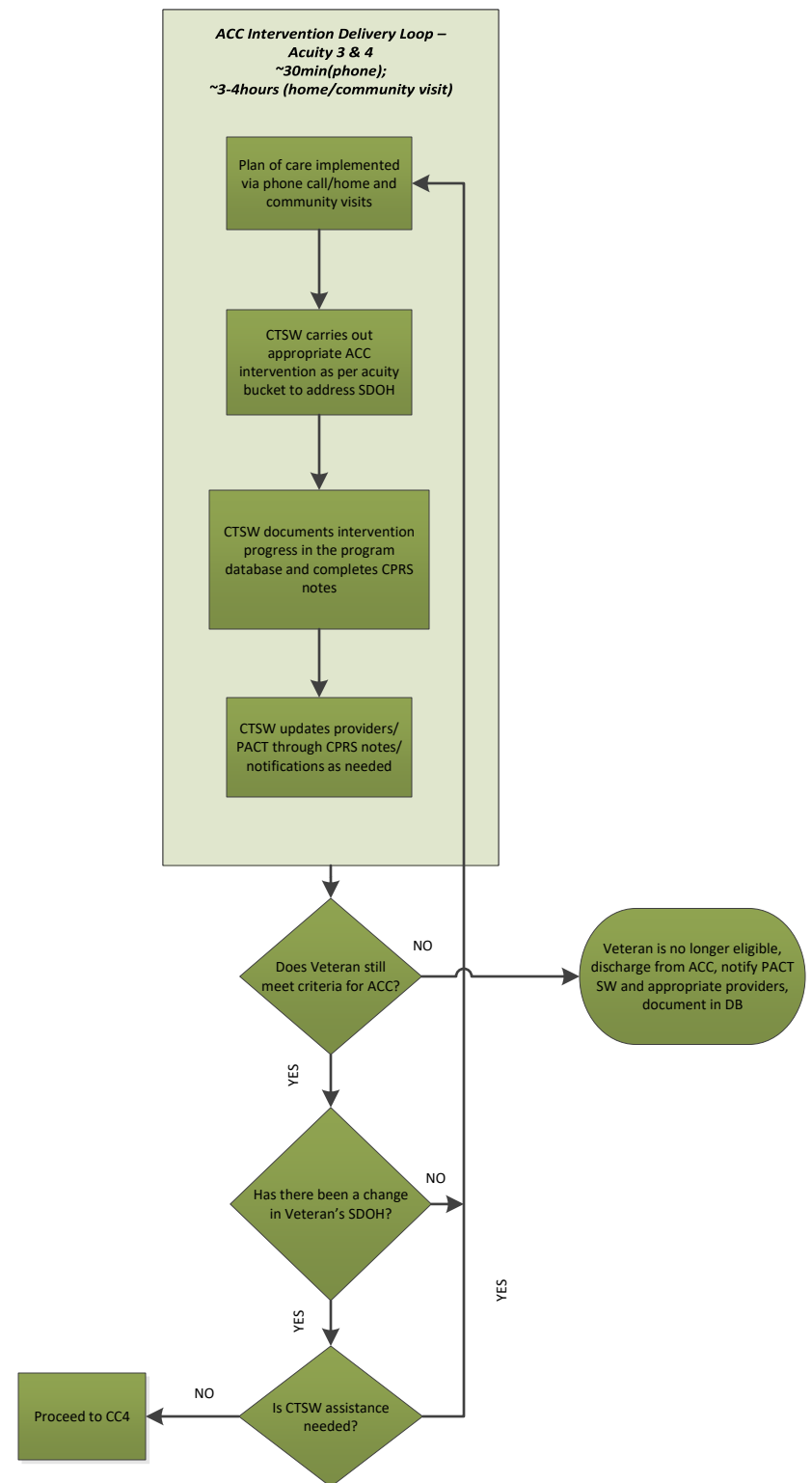

Supplement: Appendix 2 — Example of a process map. [file Data_Sheet_2.PDF]
